# Metabolomic profiling and bactericidal effect of *Polyalthia longifolia* (Sonn.) Twaites. stem bark against methicillin-resistant *Staphylococcus aureus*

**DOI:** 10.1099/acmi.0.000432

**Published:** 2023-06-29

**Authors:** Gael Tchokomeni Siwe, Marguerite Kamdem Simo, Rukesh Maharjan, Andre Perfusion Amang, Christophe Mezui, Paul Vernyuy Tan

**Affiliations:** ^1^​ Department of Animal Biology & Physiology, Faculty of Sciences, University of Yaoundé I, P.O. Box 812, Yaoundé, Cameroon; ^2^​ Department of Biological Sciences, Faculty of Sciences, University of Maroua, P.O. Box 814, Maroua, Cameroon; ^3^​ H.E.J. Research Institute of Chemistry, International Center for Chemical and Biological Sciences, University of Karachi, P.O. Box 75270, Karachi, Pakistan; ^4^​ Department of Animal Biology, Higher Teacher Training College, ENS, University of Yaoundé I, P.O. Box 47, Yaoundé, Cameroon

**Keywords:** *Polyalthia longifolia*, MRSA, bactericidal activity, LC-MS, GC-MS

## Abstract

**Objective.** The present study was carried out to establish the chemical profile of the methanolic extract of *Polyalthia longifolia* stem bark and investigate its antibacterial property against some human pathogenic bacteria.

**Methods.** The extract was analysed using liquid and gas chromatography coupled to mass spectrometry. Antibacterial activity of *P. longifolia* extract against some human pathogenic bacteria was screened using the AlamarBlue method, and MIC and MBC were determined.

**Results and Conclusion.** Liquid chromatography-mass spectrometry (LC-MS) revealed the presence of 21 compounds among which 12 were identified. Gas chromatography-mass spectrometry (GC-MS) allowed identification of 26 compounds, the three major ones being the following: cis vaccenic acid (17.79 %), 3-ethyl-3-hydroxyandrostan-17-one (13.80 %) and copaiferic acid B (12.82 %). *P. longifolia* extract was active against Gram-positive bacteria with MIC ranging from 1 to 2 mg ml^−1^ and MBC from 2 to 6 mg ml^−1^. This study demonstrated the bactericidal effect of the methanolic extract of *Polyalthia longifolia* stem bark against some human pathogenic bacteria, including methicillin-resistant *S. aureus *. This effect could be related to the presence in the extract of a broad diversity of well-known compounds with established pharmacological properties. These results support the ethnomedicinal use of *P. longifolia* stem bark in Cameroon for the management of methicillin-resistant *S. aureus* (MRSA)-related infections.

## Introduction

Although pharmaceutical industries have produced a considerable number of commercial antibiotics, resistance of pathogens to these drugs has risen to a point of global concern [1, 2]. Much like the situation in human medicine, intensive use of antibiotics in agriculture, livestock and poultry has accelerated the development of antibiotic-resistant strains, potentially complicating treatments for plants, animals and humans. Bacteria have the genetic ability to acquire and transmit resistance towards drugs to many different bacterial strains through horizontal gene transfer. Therefore, there has been an increased mortality rate due to complications of infectious diseases and treatment failure. This explains why resistance to antibiotics has been recognized by the World Health Organization as the greatest threat in the treatment of infectious diseases [3, 4]. For example, methicillin-resistant *Staphylococcus aureus* (MRSA) has been found to be the major cause of hospital-acquired infections and community-acquired infections [5]. Because of such concern, the constant need to find new antimicrobial agents is of paramount importance, alongside the necessity to seek and design alternative approaches to manage resistant bacteria. One of the possible strategies is rational valorization of bioactive phytochemicals with evidence-based antibacterial activity, as medicinal plants are important natural resources and are increasingly gaining wide spread interest for the treatment of numerous diseases.

*Polyalthia longifolia* (Sonn.) Twaites. (Annonaceae) originates from India and has been afterwards introduced to many tropical countries around the world, including Cameroon. It is an evergreen tree with a height of over 30 ft, which exhibits symmetrical pyramidal growth with willowy pendulous branches and long narrow lanceolate leaves. Due to its aesthetically pleasing structure, this tree is often used as ornamentation in households [6, 7]. Almost all parts of *P. longifolia* are used in traditional Indian medicine for the treatment of various ailments such as uterine disorders, fever, skin diseases, diabetes, hypertension, helminthiasis, gonorrhoea [8]. Several pharmacological properties were reported by researchers about the stem bark and leaves of this plant. These include antimicrobial [9], cytotoxic [10], hypotensive [11] and antiulcer [12] activities. Although numerous studies have been carried out with Asian *P. longifolia*, its antibiotic activity has not, to our knowledge, been carried out on Cameroonian *P. longifolia*. Thus, the aim of the present study was to investigate the chemical composition and to test the antibacterial effect of the stem bark extract of *P. longifolia* from Cameroon.

## Methods

### Plant material

Fresh stem bark of *Polyalthia longifolia* (Sonn.) Thwaites. were harvested in Yaoundé (Cameroon) (3.861057 N; 11.506323 E) and identified at the National Herbarium of Cameroon by comparison with existing voucher number 67474HNC. The stem barks were chopped into small pieces, shade-dried and then crushed, using an electric blender to obtain a fine powder.

### Extract preparation

In total, 100 g of *Polyalthia longifolia* stem bark powder was macerated in 3 l of methanol for 48 h at room temperature. The mixture was filtered through Whatman filter paper № 3 and the resulting filtrate was then concentrated at (40 °C) using a rotative evaporator (Heidolph, Germany). The paste obtained was dried in a convection oven (Jencons-PLS, UK) set at 40 °C. The resulting extract [5.3 g (5.3 % yield)] was stored at 4 °C for later use.

### Bacterial strains

Ten (10) bacterial strains were used in this study. Gram-positive bacteria were *Staphylococcus aureus* (ATCC 6538), *Enterococcus faecalis* (ATCC 39532), *Bacillus subtilis* (ATCC 23857), *Streptococcus oralis* (ATCC 10557), methicillin-resistant *Staphylococcus* aureus (NCTC 13277) and methicillin-resistant *Staphylococcus* aureus (NCTC 13143). Gram-negative bacteria included *Pseudomonas aeruginosa* (ATCC 10145), *Salmonella typhi* (ATCC 14028), *Escherichia coli* (ATCC 25922) and *Helicobacter pylori* (clinical isolate). Bacterial strains were obtained from the microbial bank of the International Center for Chemical and Biological Sciences (ICCBS), University of Karachi, Pakistan.

### Gas chromatography-mass spectrometry analysis

The GC-MS characterization of the methanolic extract of *Polyalthia longifolia* stem bark was done using Agilent Technologies GC systems with GC-7890A/MS-5975C models (Agilent Technologies, Santa Clara, CA, USA) equipped with HP-5MS column (30 m in length ×0.32 mm in diameter ×0.25 µm in thickness of film). After solubilization in methanol, the extract was filtered through a 0.45 µm filter (Millipore, USA) and then injected in splitless mode. Spectroscopic detection by GC-MS involved an electron ionization system, which utilized high-energy electrons (70 eV). The carrier gas was helium with a flow rate of 1.0 ml min^−1^. The initial temperature was set at 50 °C with increasing rate of 5 °C min^−1^ and holding time of about 10 min. Finally, the temperature was increased to 300 °C at 10 °C min^−1^. The compounds were identified by comparison of their mass spectra with standards available in National Institute of Standards and Technology (NIST) (Gaithersburg, USA) mass spectral library.

### Liquid chromatography-mass spectrometry analysis

LC-MS characterization of the methanolic extract of *Polyalthia longifolia* stem bark was realized using a Hewlett-Packard 1100 chromatograph (Agilent Technologies, Santa Clara, CA, USA) with diode array detector (DAD), and equipped with a C_18_ column (4.6 mm × 150 mm × 5 µm particle size). The mobile phases were 0.1 % formic acid in water (A) and 0.1 % formic acid in methanol (B) at a flow rate of 0.5 ml min^−1^. The chromatographic method consisted of the following elution gradient: 10 % B for 1.00 min; 10–100 % B for 6.00 min, 100 % B for 1.0 min. An MSD Ion Trap XCT mass spectrometer (Agilent Technologies, Santa Clara, CA) equipped with an electrospray ionization (ESI) interface was used in positive ionization mode (between 100 and 1200 m/z) for the MS analysis using data-dependent automatic switching between MS and MS/MS acquisition modes. Identification of compounds was conducted using a cross comparison of their mass spectra with standards contained in three online spectral databases, namely, spectral database for organic compounds (SDBS) (sdbs.db.aist.go.jp), mass bank Europe (massbank.eu) and metlin database (metlin.scripps.edu).

### Bacterial susceptibility testing procedure (Microplate AlamarBlue assay)

The microplate AlamarBlue assay was used to check the antibacterial activity of *Polyalthia longifolia* against the bacterial strains under study. This assay is based on resazurin dye (7-hydroxy-10-oxidophenoxazin-10-ium-3-one), which is an oxidation-reduction (REDOX) weakly fluorescent indicator that undergoes colorimetric change (from blue to pink/red) in response to cellular metabolic reduction. Its reduced form, resorufin, is pink and highly fluorescent, and the intensity of fluorescence produced is proportional to the number of living cells. One colony of each of the aforementioned strains (except *Helicobacter pylori *), grown in Tryptone Soya Agar (TSA) (Oxoid, UK), was inoculated in Mueller–Hinton Broth (MHB) (Oxoid, UK) and incubated at 37 °C for 22 h. Bacterial cultures obtained were diluted using MHB and adjusted to 0.5 McFarland turbidity index (~1.5×10^8^ c.f.u. ml^−1^). A stock solution (40 mg ml^−1^) of *P. longifolia* extract was prepared in 10 % DMSO and 10 µl of this stock solution were placed in all the wells of a sterile 96-well microplate except positive (bacteria+medium), negative (medium +10 % DMSO) and drug (bacteria+medium+standard antibiotics) control wells. After adding appropriate volume of MHB in all the wells, bacterial suspensions (5 µl) were then introduced, except in negative control wells. The concentration of extract in the final 200 µl solution was 2 mg ml^−1^. Tetracycline was used as the control drug with a stock solution prepared at 1 mg ml^−1^ in distilled water. After 22 h of incubation at 37 °C, 20 µl of 0.02 % AlamarBlue dye (Chem-Impex-International, USA) solution was added in all the wells, and the plates incubated at 37 °C in a shaking incubator at 80 r.p.m. for 2 h. *Helicobacter pylori* was cultured in Brain Heart Infusion Agar (BHIA) supplemented with Laked Horse Blood (Oxoid, UK), and *H. pylori* selective supplement (Dent) (Oxoid, UK). Bacterial suspensions were diluted in Brain Heart Infusion Broth (BHIB) and incubated for 48 h at 37 °C in 10 % CO_2_ incubator. Amoxicillin and metronidazole, prepared at 1 mg ml^−1^ each, were used in mixture (1 : 1) as the control drug. For quantitative analysis, absorbance was read at two wavelengths (570 and 600 nm) using a spectrophotometer (Thermo Scientific, USA). The percentage of inhibition of bacterial growth due to extract/control drug treatment was calculated as described by Al-Nasiry *et al*. [13]. Each experiment was run in triplicate.

### Determination of minimum and maximum inhibitory concentrations

MIC was determined using the standard broth microdilution method in accordance with the Clinical and Laboratory Standards Institute (CLSI) guidelines [14]. *P. longifolia* extract was serially diluted twofolds in MHB with final concentrations ranging as follows: 0.062, 0.125, 0.25, 0.50, 1, 2, 4 and 8 mg ml^−1^. Thereafter, 5 µl of respective bacterial suspensions (except *Helicobacter pylori *) prepared as described above, were added in all the wells, and plates incubated for 22 h at 37 °C. For *H. pylori *, MHB was replaced by BHIB. The lowest concentration of extract, capable of inhibiting visible growth with no turbidity, was then recorded as the MIC.

Minimal bactericidal concentration (MBC) was determined by sub-culturing 50 µl of each well content, from serial dilutions described above, in TSA plates. TSA plates were incubated at 37 °C for 22 h. For *H. pylori *, TSA plates were replaced by BHIA supplemented with Laked Horse Blood (Oxoid, UK), and *H. pylori* selective supplement (Dent) (Oxoid, UK). The lowest concentration of extract without any bacterial growth was recorded as MBC. The experiments were carried out in triplicate for each bacterial strain.

## Results

### GC-MS analysis

**Fig. 1.**
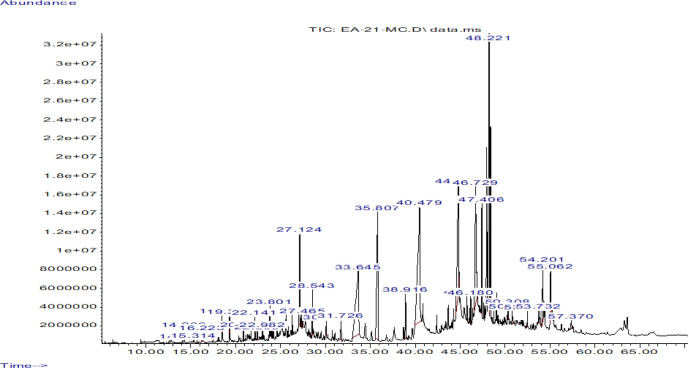
GC-MS spectra of the methanolic extract of *P. longifolia* stem bark.

The GC-MS spectra (Fig. 1) allowed identification of 26 compounds being reported, alongside their retention time, monoisotopic mass and abundance (peak area %), in Table 1.

**Table 1.** Phytoconstituents identified by GC-MS from methanolic extract of *P. longifolia*

	Names	Formula	Monoisotopic mass	retention time (min)	Area (%)
1	3,5-dihydroxy-6-methyl-2,3-dihydro-4H-pyran-4-one	C6H8O4	144.042	14.23	0.43
2	Dihydrocoumarone	C8H8O	120.057	16.22	0.41
3	2-methoxy-4-vinylphenol	C9H10O2	150.068	18.46	0.68
4	Syringol	C8H10O3	154.063	19.32	0.56
5	Curcumene	C15H22	202.172	22.14	0.86
6	3',5′-dimethoxyacetophenone	C10H12O3	180.079	23.80	0.63
7	Longipinocarveol, trans-	C15H24O	220.183	26.28	0.42
8	coniferol	C10H12O3	180.079	27.12	3.4
9	9,10-epoxycedrane	C15H24O	220.183	28.54	1.27
10	Corymbolone	C15H24O2	236.177	30.08	0.52
11	Methyl palmitate	C17H34O2	270.256	31.73	0.84
12	Hexadecanoid acid	C16H32O2	256.240	33.64	11.55
13	12-Isopropyl-1,5,9-trimethyl-4,8,13-cyclotetradecatrien-1,3-diol	C20H34O2	306.256	35.81	11.24
14	Methyl 11-octadecenoate	C19H36O2	296.271	38.91	1.68
15	cis-vaccenic acid	C18H34O2	282.256	40.48	17.79
16	Copaiferic acid B	C20H32O2	304.240	44.80	12.82
17	Heptatriacontan-1-ol	C37H76O	536.589	45.76	0.51
18	5-(7a-isopropenyl-4,5-dimethyl-octahydroinden-4-yl)−3-methyl-pent-2-en-1-ol	C20H34O	290.261	46.73	10.04
19	β-levantenolide	C20H30O3	318.46	47.40	2.49
20	3-ethyl-3-hydroxyandrostan-17-one	C21H34O2	318.256	48.22	13.80
21	11-hydroxy-7,8-epoxylanostan-3-yl acetate	C32H54O4	502.402	50.31	0.83
22	Astaxanthin	C40H52O4	596.838	50.78	0.53
23	Vitamin E	C29H50O2	430.381	52.48	0.38
24	Campesterol	C28H48O	400.680	53.73	0.40
25	Stigmasterol	C29H48O	412.370	54.20	2.88
26	Clionasterol	C29H50O	414.386	55.06	3.04

**Fig. 2.**
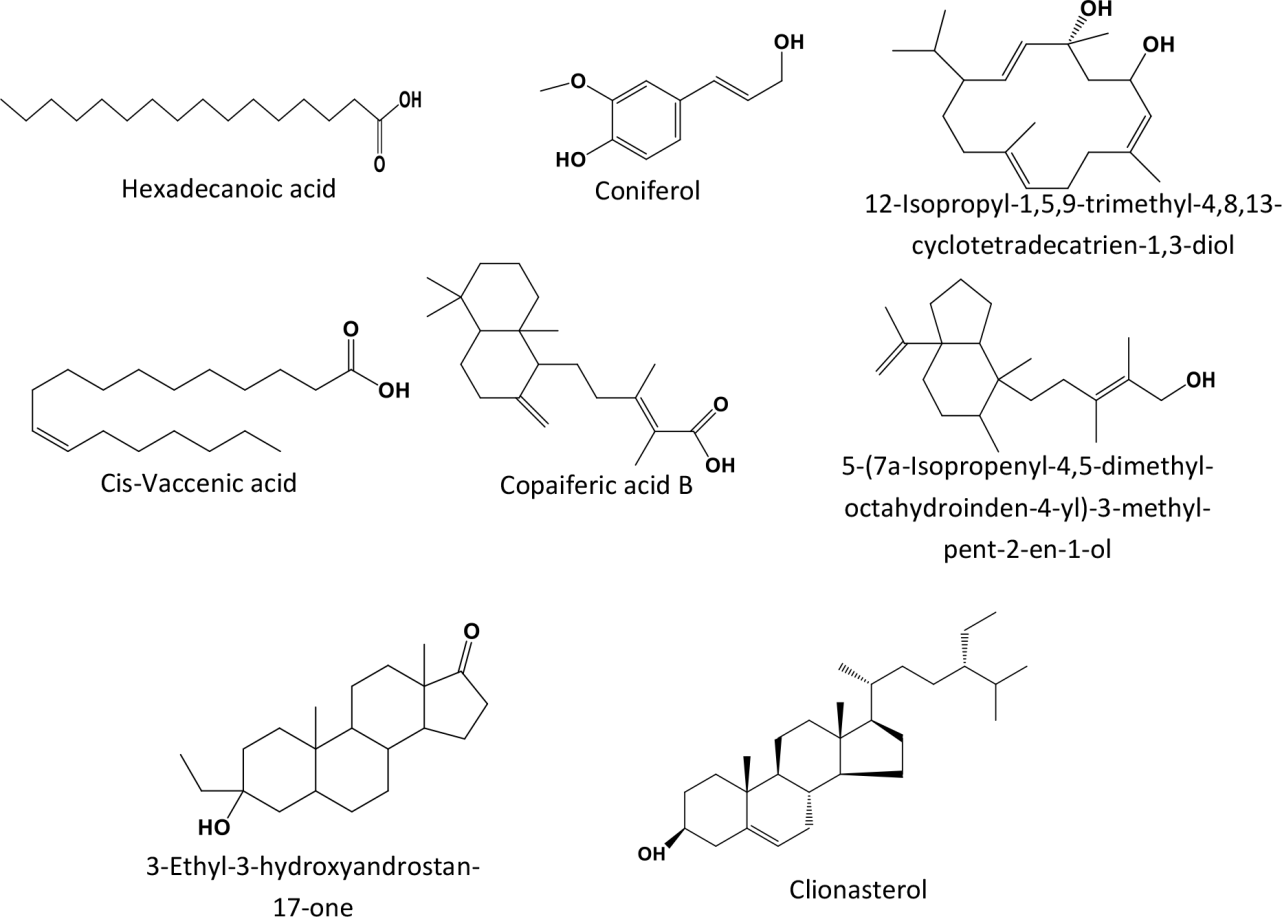
Structures of major compounds identified by GC-MS.

Structures of the eight (8) most abundant compounds, namely: cis-vaccenic acid (17.79 %), 3-ethyl-3-hydroxyandrostan-17-one (13.80 %), copaiferic acid B (12.82 %), palmitic acid (11.55 %), 12-isopropyl-1,5,9-trimethyl-4,8,13-cyclotetradecatrien-1,3-diol (11.24 %), 5-(7a-isopropenyl-4,5-dimethyl-octahydroinden-4-yl)−3-methyl-pent-2-en-1-ol (10.04 %), coniferol (3.4 %), and clionasterol (3.04 %), are depicted in Fig. 2.

### LC-MS analysis

**Fig. 3.**
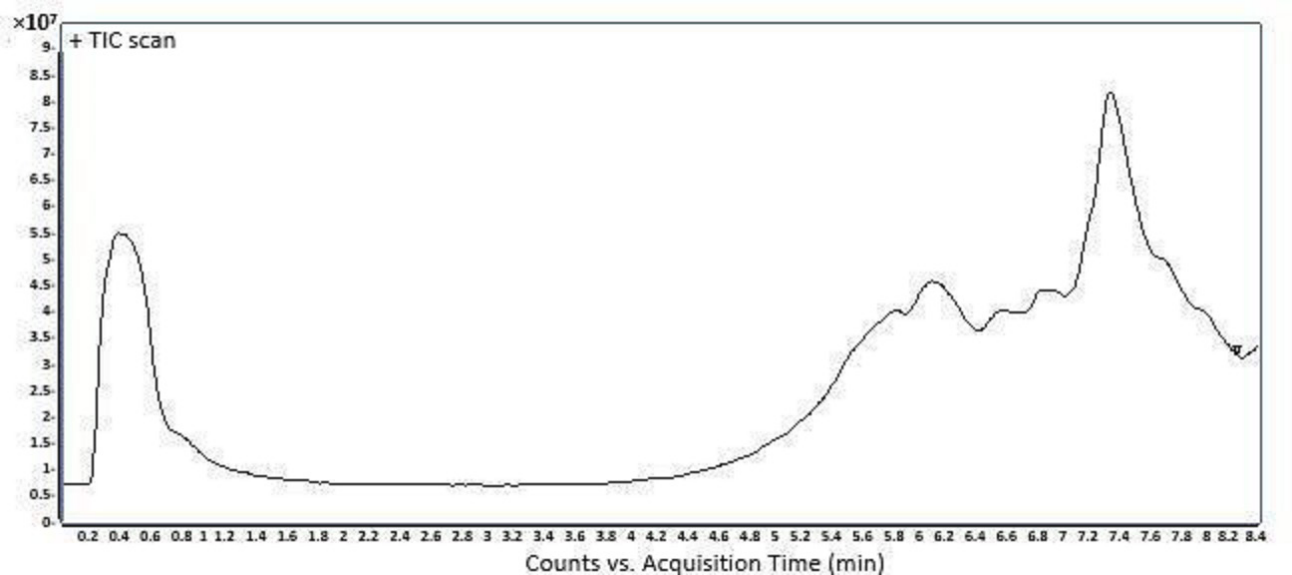
Total ion chromatogram of the methanolic extract of *P. longifolia* stem bark.

**Fig. 4.**
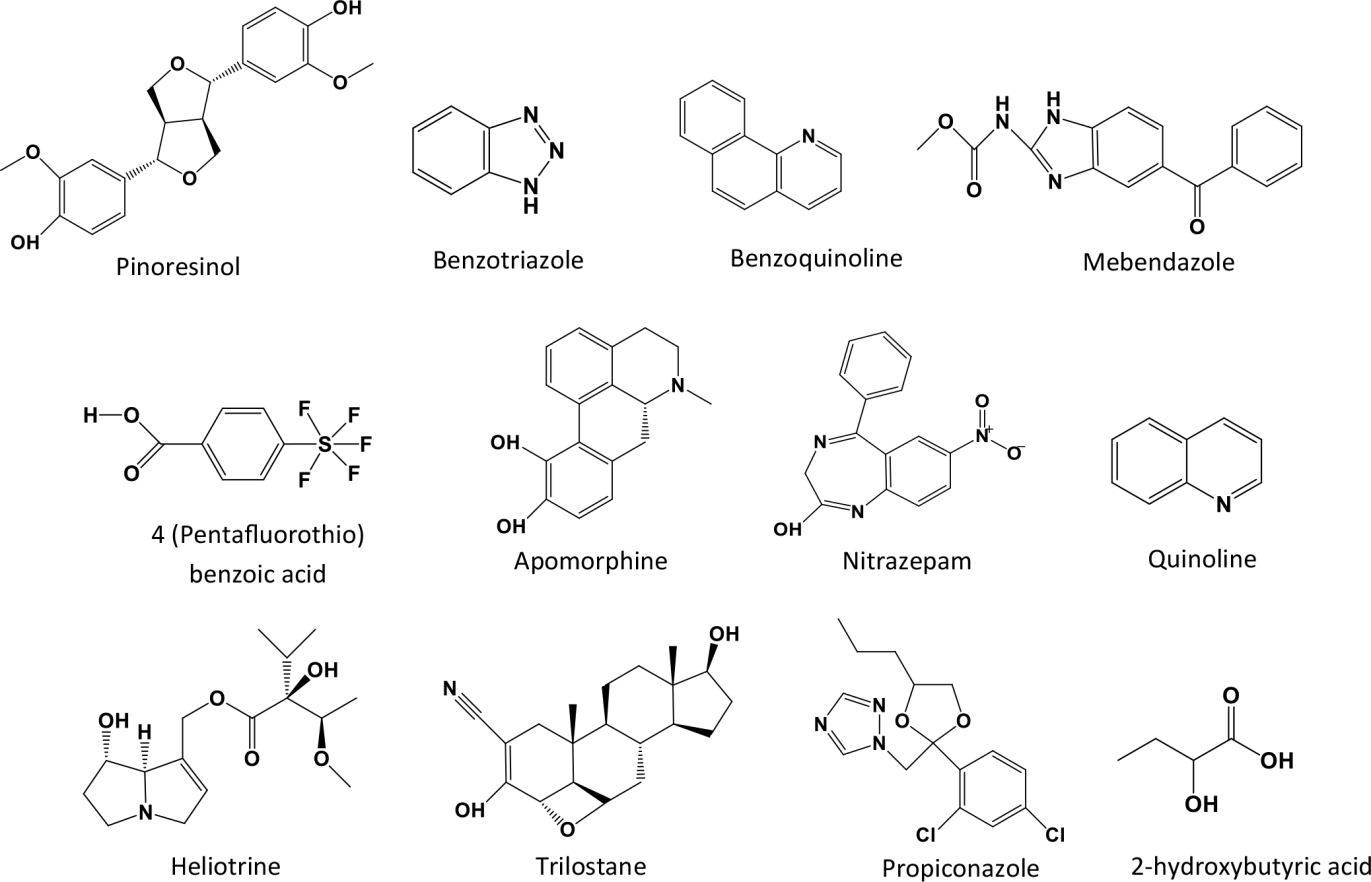
Structures of compounds identified by LC-MS.

The total ion chromatogram (TIC) of *P. longifolia* methanolic extract is represented by Fig. 3. Twelve (12) compounds were identified and summarized in Table 2, with their chemical structures illustrated by Fig. 4.

**Table 2.** Phytoconstituents identified by LC-MS from methanolic extract of *P. longifolia*

№	Formula	Monoisotopic mass	Tentative identification	Retention time (min)
1	C4H8O3	104.0473	2-hydroxybutyric acid	0.352
2	C6H5N3	119.0483	Benzotriazole	0.378
3	C_13_H_9_N	179.0735	Benzoquinoline	0.391
4	C9H7N	129.0578	Quinoline	0.426
5	C7H5F5O2S	247.9930	4 (Pentafluorothio)benzoic acid	0.483
6	C_17_H_17_NO_2_	267.1259	Apomorphine	0.497
7	C15H11N3O3	281.0800	Nitrazepam	0.546
8	C_16_H_13_N_3_O_3_	295.0957	Mebendazole	0.608
9	C16H27NO5	313.1889	Heliotrine	0.684
10	C_20_H_27_NO_3_	329.1990	Trilostane	5.188
11	C_15_H_17_Cl_2_N_3_O_2_	341.0697	Propiconazole	5.348
12	C20H22O6	358.1416	Pinoresinol	6.983

### Antibacterial activity

Antibacterial activity of *P. longifolia* methanolic extract against Gram-positive and Gram-negative bacteria is described in Tables 3 and 4, respectively. Percentages of inhibition were ranged from 79.05–97.81 % (except *Enterococcus faecalis *) for Gram-positive bacteria and from 1.76–7.64 % for Gram-negative bacteria.

**Table 3.** Activity of *P. longifolia* (% inhibition) against Gram-positive bacteria

Treatment	* S. aureus *	* E. faecalis *	* B. subtilis *	* S. oralis *	Resistant * S. aureus* (13277)	Resistant * S. aureus* (14143)
*P. longifolia*	97.81±2.16	30.74±1.22***	92.24±3.48	79.05±1.84	96.16±2.54	97.26±1.54
Tetracycline	85.85±2.00	87.12±0.71	92.74±2.41	89.46±1.67	85.23±2.08	84.98±1.65

Values are presented as mean percentage ±sem of three replicates. *P. longifolia* extract was tested at 2 mg ml^−1^. ***P<0.001=significant statistical difference by comparison with tetracycline.

**Table 4.** Activity of *P. longifolia* (% inhibition) against Gram-negative bacteria

Treatment	* P. aeruginosa *	* S. typhi *	* E. coli *	* H. pylori *
*P. longifolia* (2 mg ml^−1^)	4.25±0.05***	7.64±1.07***	6.87±0.08***	1.76±0.04***
Tetracycline	87.12±1.48	84.77±1.97	85.48±1.11	na
Amoxicillin+metronidazole	na	na	na	79.08±2.18

Values are presented as mean percentage ±sem of three replicates. Amoxicillin and metronidazole in combination were tested at 25 µg ml^−1^ each. ***P<0.001=significant statistical difference by comparison with tetracycline. NA=non applicable.

MIC and MBC of *P. longifolia* methanolic extract against some Gram-positive bacteria are presented in Table 5. MIC values were comprised between 1000 µg ml^−1^ and 2000 µg ml^−1^, and MBC values between 2000 and 6000 µg ml^−1^.

**Table 5.** MIC and MBC values (μg ml^−1^) of *P. longifolia*

	* S. aureus *	* S. oralis *	* B. subtilis *	Resistant *S. aureus* (13277)	Resistant *S. aureus* (14143)
MIC	1000	2000	1000	1000	1000
MBC	2000	6000	4000	4000	4000

## Discussion

The methanolic extract of *P. longifolia* stem bark showed good antibacterial activity against Gram-positive bacteria, irrespective of whether they were sensitive or methicillin-resistant strains, and was ineffective on Gram-negative bacteria. In Gram-positive bacteria, the MBC/MIC ratio was found to be less than 4, which classifies the extract as bactericidal against these bacteria as per the Marmonier [15] recommendations. The difference in sensitivity to Gram-negative and Gram-positive bacteria may be due to the variation in their cell-wall structure. Most Gram-positive bacteria are surrounded by a coarse peptidoglycan cell wall. The Gram-positive bacterial cell wall consists of 70–100 layers of peptidoglycans, which are comprised of two polysaccharides, N-acetyl-glucosamine and N-acetyl-muramic acid, cross-linked by peptide side chains and cross bridges. This structure, although mechanically strong, appears to offer little resistance to the diffusion of small molecules such as antibiotics. In contrast, Gram-negative bacteria, surround themselves with a second membrane, the outer membrane, which functions as an effective barrier [16, 17]. In a study conducted by [9], the methanolic extract of *P. longifolia* leaves, harvested in Karachi (Pakistan), exhibited good antibacterial activity against both Gram-positive and Gram-negative bacteria. MIC values were ranging from 125 µg ml^−1^ (* Pseudomonas aeruginosa *) to 500 µg ml^−1^ (* Salmonella typhi *) for Gram-negative bacteria, and 125 µg ml^−1^ for the three Gram-positive bacteria tested. On the other hand, another study [18] investigating antimicrobial activity of the methanolic extract of *P*. *longifolia* leaves, harvested in India, reported MICs of 3125 µg ml^−1^ against *Staphylococcus aureus *, and higher than 25 000 µg ml^−1^ against *Salmonella typhimurium *. These results are closer to those of the present study, with both extracts seeming less active that the one harvested in Karachi, Pakistan. This difference between the results of the Karachi study and those of the present study could be due to a plethora of factors among which an uneven distribution of active metabolites between the plant’s organs and/or between plants located in different ecological zones. These factors could include seasonality, temperature, water availability, UV radiation, nutrients present in soil, altitude, air pollution and even induction by mechanic stimuli or attack by pathogens.

LC-MS analysis allowed identification of 12 compounds, some with tested antibacterial properties. For example, heliotrine has been reported to be effective against bacteria (* E. coli *, *S. pneumoniae *, *B. subtilis* and *B. anthracis *) and fungi (*Aspergillus fumigatus*, *Aspergillus niger* and *Penicillium chrysogenum*) [19]. Heliotrine-N-oxide is a pyrrolizidine alkaloid (PA) and pyrrolizidine alkaloids are secondary metabolites, produced by several plant species as protection against insect herbivores. The PA-content depends on several factors (species, plant organ, harvest, storage, extraction procedures). Contents vary from trace amounts up to 19 % based on dry weight. A study by Vrieling *et al*. [20] using a diallel cross of *Senecio jacobaea* revealed that 48 % of the variation in total PA content was caused by genotypic variation. This variability is further increased by the possible formation of monoesters at different positions and open or cyclic diesters. The effect of each single PA is dependent on its chemical structure and physical properties such as lipophilicity, hydrophilicity and pharmacokinetics [20]. These reasons may explain a possible low level of heliotrine in the whole extract, and thus, a decreased potency against Gram-negative bacteria in the present study. Apomorphine was found to be bactericidal against *E. coli* by targeting the pleiotropic bacterial regulator Hfq [21]. Pinoresinol showed good antibacterial activities against most of bacteria studied in the current work [22], while propiconazole and mebendazole demonstrated their potency against several fungi strains including *Cryptococcus neoformans* and *Sclerotium rolfsii* [23, 24]. Among the 26 compounds identified by GC-MS, some were equally reported in the literature for their antimicrobial properties. For instance [25], demonstrated antibacterial potential of coniferol against *E. coli* and *B. cereus *, while the bactericidal effect of hexadecenoic acid against *S. aureus* was also evidenced [26]. All these compounds present in the methanolic extract of *P. longifolia* stem bark, and possibly acting either synergistically or additively, could be responsible for the observed bactericidal effect of this extract. Antibacterial activity of plants is directly linked to the diversity and complexity of their secondary metabolites. Major groups of phytochemicals, which are known to possess antimicrobial properties are the following: polyphenols (flavonoids, quinones, tannins, coumarins), terpenoids, alkaloids, lectins and polypeptides [27]. Therefore, the relatively high MIC and MBC values of *P. longifolia* stem bark extract could be explained by its low content of bioactive compounds such as heliotrine, apomorphine or pinoresinol.

## Conclusion

LC-MS and GC-MS analysis of methanolic extract of *P. longifolia* stem bark permitted the identification of valuable compounds with well-established antibacterial properties, which would be responsible of the extract’s bactericidal activity. These compounds would act either synergistically or additionally to produce the bactericidal effect. Further studies are planned to investigate the underlying mechanisms involved to this end.
